# The Simultaneous Use of 1-Methylcyclopropene and Methyl Jasmonate Vapor as an Innovative Strategy for Reducing Chilling Injury and Maintaining Pomegranate Fruit Quality at Suboptimal Temperatures

**DOI:** 10.3390/foods13010060

**Published:** 2023-12-22

**Authors:** José Manuel Lorente-Mento, María Serrano, Domingo Martínez-Romero, María Celeste Ruiz-Aracil, Daniel Valero, Fabián Guillén

**Affiliations:** Postharvest Research Group of Fruit and Vegetables, CIAGRO, University Miguel Hernández, Ctra. Beniel km. 3.2, 03312 Orihuela, Alicante, Spain

**Keywords:** chilling injury, elicitor, 1-MCP, *Punica granatum* L., quality, storage

## Abstract

Spain is one of the main contributors to global pomegranate production. Pomegranate presents a challenge for preservation at suboptimal temperatures. Preserving this fruit for an extended period is challenging due to its susceptibility to chilling injury (CI). For this reason, we have examined different postharvest treatments to extend the pomegranate shelf life and their potential impact on reducing CI. For this reason, two postharvest treatments have been applied: 1-Methylcyclopropene (1000 nL L^−1^ 1-MCP) and methyl jasmonate vapors (0.01 mM MeJA), a natural elicitor found in many plant organs that induces a wide range of physiological processes, including the activation of defense mechanisms against stress. Following the application of these treatments and subsequent fruit storage at 2 °C for 90 days, maintenance of firmness and membrane integrity was observed. Additionally, a positive synergic effect was observed in these quality traits when combining both substances (1-MCP + MeJA), especially with regard to delaying weight loss, the external color evolution, and total polyphenol accumulation. On the other hand, MeJA treatment alone or in combination with 1-MCP also increased the anthocyanin content in arils, thereby enhancing the fruit quality. In general, the best results were observed when these two different technologies were applied as a combined treatment, especially in terms of maintaining quality traits such as fruit firmness and total acidity and reducing weight loss and CI. This is the first time that these two substances have been tested together in any fruit species, and their simultaneous application in the same container represents an innovative approach that could be an interesting tool for commercial purposes.

## 1. Introduction

Pomegranate (*Punica granatum* L.) fruit is a plant species that originated in the Middle East but has spread throughout the Mediterranean and other countries over the years [1]. Currently, pomegranate is widely valued by consumers primarily for its sensory qualities and the existence of beneficial compounds like flavonoids, anthocyanins, and tannins. Scientific research has revealed that these compounds, particularly with their antioxidant properties, can contribute positive effects to human health and could be used as a preventive strategy for different diseases, such as cardiovascular diseases, diabetes, cancer, etc. [2,3]. Pomegranate fruit, once harvested, continues its metabolic activity, leading to different alterations that cause a decrease in quality. Among the most frequent alterations are dehydration, increased respiratory rate, and rotting. Currently, there are different technologies that help to delay pomegranate fruit senescence, such as cold storage, which prolongs fruit quality during its storage. However, temperatures below 5 °C cause pomegranate chilling injury (CI) [4], which results in discoloration and pitting of the pomegranate peel and aril browning. In this sense, CI contributes to a greater presence of microorganisms and, therefore, to the rot incidence [5]. In recent years, different methods or strategies have been used and/or studied to help minimize the presence of CI in pomegranate fruit. For example, it has been observed that physical barriers such as packaging or wrapping with different films successfully maintain quality traits during refrigerated storage [6] and also reduce CI at suboptimal temperatures [7]. With respect to chemical treatments, salicylic acid (SA) has the potential to decrease both ethylene production and the respiration rate, maintaining pomegranate quality during postharvest storage at low temperatures [8]. In this sense, SA stimulates CI tolerance by activating antioxidant systems and increasing resistance to different pathogens [9]. Other chemical substances such as methyl salicylate, γ-aminobutyric acid (GABA), or glycine betaine were able to reduce CI in pomegranate, orange, and kiwifruit during storage [10,11,12]. Other strategies as intermittent warming, controlled atmosphere, or the application of nitric oxide have demonstrated success in controlling CI [13]. Methyl jasmonate (MeJA) is an elicitor that stimulates plant growth and development and is involved in the plant defense system against several biotic and abiotic stresses [14]. For this reason, it has been applied to maintain fruit quality stored at suboptimal temperatures [15]. Additionally, in previous studies we have observed that MeJA solutions applied at pre- and postharvest reduced CI in the pomegranate rind when stored at 2 °C, which has been linked to higher stability of the cell membranes [16]. It has also been shown that MeJA solutions improve the antioxidant activity in pomegranate arils [17,18] and other non-climacteric fruit, such as strawberries stored at 1 °C [19]. On the other hand, blocking ethylene action with 1-methylcyclopropene (1-MCP) can reduce fruit senescence after harvest as well as CI [20]. Similar effects were observed after applying 1-MCP to kiwifruit [21]. Recently, different studies displayed a synergistic effect between the combination of 1-MCP with different plant-origin substances such as salicylic acid derivatives, essential oils, or melatonin [22,23,24]. For example, several studies evaluating the effect of MeJA treatments in comparison with 1-MCP obtained positive effects with both technologies in different fruit species such as kiwifruit and peach [25,26]. Nevertheless, to the best of our knowledge, this is the first time in which a combined treatment with these two different fumigation technologies has been applied to evaluate CI in any fruit species. For this reason, in this study, we have evaluated the potential of these technologies, combined or separately, on pomegranate fruit to assess their impact on fruit shelf life and against CI during cold storage.

## 2. Materials and Methods

### 2.1. Fruit Material and Postharvest Treatments

The harvested fruit (*Punica granatum* L. cv. Mollar de Elche) were selected in accordance with the size homogeneity, external color, and soluble-solid-level (14–15° Brix) characteristics of this variety. In preliminary experiments, 0.01, 0.1, or 1 mM MeJA vapor applications were assayed alone or in combination with 1-MCP [27], and the most effective conditions that reduced CI symptoms and delayed other quality parameters (0.01 mM + 1-MCP) were selected. For this reason, in the present study, MeJA treatment (0.01 mM) was assayed alone or in combination with 1-MCP (1000 nL L^−1^) for 24 h under room temperature conditions, as recommended by Li et al. [26] for 1-MCP treatments in pomegranate fruit. The application of 1-MCP treatments was conducted by combining tablets available commercially with an activator solution. All these chemicals were supplied by SmartFresh (Agro-Fresh Inc., Philadelphia, PA, USA). Treatments were assayed in 3 replicates, using batches of 15 pomegranates containing 5 fruits per replicate and sampling time. The different lots were placed inside distinct sealed containers. For MeJA treatments, the necessary volume of this substance required to achieve the desired concentration was placed on a filter paper at the container’s bottom, and the container was immediately closed so that it was airtight. MeJA treatments for each replicate were applied in the vapor phase for 24 h at 20 °C either alone or combined with 1-MCP (1000 nL L^−1^). Another batch without treatment was stored in similar containers under the same conditions (Control). After the treatments, the pomegranate fruit were removed and transferred to suboptimal temperatures (2 °C) and 85% RH to trigger CI [10]. Samples were studied over a period of 90 days in periods of 30 days of refrigerated storage plus 3 days at room temperature.

### 2.2. Postharvest Quality Parameters

Respiration rate was measured in a secure airtight 3.7 L container for a duration of 60 min, using 5 fruits per replicate and treatment. Subsequently, a 1 mL gas sample was extracted from the headspace, and carbon dioxide levels were measured using a Shimadzu 14B gas chromatograph (Shimadzu Europa GmbH, Duisburg, Germany) equipped with a thermal conductivity detector. The chromatographic parameters have been detailed in previous studies by Martínez-Romero et al. [28]. This parameter was elucidated as mg CO_2_ kg^−1^ h^−1^. The weight loss of pomegranates was evaluated and calculated in relation to the fruit weight at the beginning of the experiment. Fruit softening was measured in each fruit, using a flat probe in a TX-XT2i texture analyzer (Stable Microsystems, Godalming, UK), applying the necessary force to achieve a 5% deformation of the fruit diameter. The obtained results were displayed as the ratio of the applied force to the distance covered (N mm^−1^), presented in this study as the mean ± standard error (ES). Outer-rind color parameters were evaluated individually at three different areas across the equatorial area of 5 fruits per replicate. Color evaluations were assayed for each pomegranate on the skin (external) and arils using a colorimeter (CRC400, Minolta Camera Co., Tokyo, Japan). CIE hue angle* (180 + tan^−1^ b*/a*, if a*< 0) results were displayed as the mean ± SE (*n* = 3) [17]. Then, after cutting the pomegranate fruit in half, 3 simultaneous measurements were collected on the aril surface for each pomegranate individually to evaluate the internal color. Thereafter, the skin and arils were taken separately from all the fruits in each replicate and mixed homogeneously. Evaluation of the total content of soluble solids (TSS) from each replicate was performed in duplicate with a refractometer at 20 °C (Atago PR-101, Atago Co., Ltd., Tokyo, Japan) by squeezing the arils obtained from the 5 fruits of each replicate, using two layers of cotton cloth. This filtered and homogenized juice of each replica was evaluated, and the results were displayed in g 100 g^−1^. In each of these juices, the titratable acidity (TA) was also evaluated. The evaluations were measured in duplicate by titration in 1 mL of diluted (25 mL) juice (785 DMP Titrino, Metrohm). The registered observations were displayed as g malic acid equivalent per 100 g^−1^ as described for pomegranates by García-Pastor et al. [17].

### 2.3. Bioactive Compounds

To determine the polyphenol content, 5 g of arils or 5 g of rind from each sample per replicate were mixed and blended in 15 mL of methanol:water (8:2) containing 2 mM sodium fluoride (with the aim of deactivating polyphenol oxidase enzyme activity and preventing polyphenol breakdown) for 60 s with a homogenizer. Extractions were subjected to centrifugation (10,000× *g*) at 4 °C for 15 min. The supernatant was evaluated with the Folin–Ciocalteu solution in duplicate for each replica to quantify the total phenolic content, following the methodology previously reported by García-Pastor et al. [17]. These authors also described the method to evaluate the anthocyanin content. These bioactive compounds were obtained by extraction from 5 g of arils and homogenization for 1 min in 15 mL of methanol/formic acid/water (80:1:19) solution with a homogenizer (Ultraturrax, T18 basic, IKA, Berlin, Germany). The extracted samples were subjected to centrifugation (10,000× *g*) at 4 °C for a duration of 15 min. Finally, the anthocyanin content was evaluated in the supernatant in duplicate at 520 nm (UV-1900i, Shimadzu, Duisburg, Germany). The total anthocyanin content was expressed as cyanidin 3-glucoside equivalents (molecular weight of 449.2 g mol^−1^ and molar absorption coefficient of 23,900 L cm^−1^ mol^−1^) and represent the mean ± SE (*n* = 3).

### 2.4. Membrane Permeability and Cold Damage

Malondialdehyde (MDA) content has been evaluated in pomegranate rind tissue, as reported by Zhang et al. [29]. Using a mortar and pestle, a 2.5 g sample was mixed and blended with 10 mL of a 10% trichloroacetic acid solution. Subsequently, supernatants were obtained after centrifuging the extract at 4 °C (10,000× *g*) for a duration of 20 min. Six mL of 0.67% thiobarbituric acid was shaken with a vortex in a test tube containing part of the supernatant (2 mL). All the tubes were subsequently immersed in a water bath set at 95 °C. After 20 min in the water bath, samples were allowed to cool at room temperature and studied using spectrophotometry (1900 UV/Vis, Shimadzu, Kyoto, Japan) at 450, 532, and 600 nm. Each evaluation was performed in duplicate, and the MDA content was evaluated as μmol kg^−1^. Membrane permeability was estimated with the method described by Yuan et al. [24] for evaluating electrolyte leakage (EL). From each treatment group, 45 disks (15 disks per replicate) with a diameter of 1 cm were obtained from the pomegranate rind. Briefly, the pomegranate disks were subjected to three successive rinses for 3 min each in deionized water. Subsequently, the disks were immersed in deionized water (50 mL) at ambient temperature for 4 h under constant shaking. After this time, the electrical conductivity (C_1_) at 20 °C in these solutions was evaluated. Samples were frozen for 24 h and then exposed to high temperature (121 °C) for 15 min. All the samples were allowed to cool, and the final electrical conductivity (C_2_) was measured at 20 °C. Electrolyte leakage (EL) was evaluated using the formula EL = (C_1_/C_2_) × 100. CI to the outer and inner rind of the pomegranate was assessed visually as we recently described [7], with 9 trained evaluators scoring the affected pomegranate surface. Scores were displayed on a 5-point scale: 1 = no damage; 2 = 1–10% surface damaged; 3 = 11–30% surface damaged; 4 = 31–50% surface damaged; 5 = >50% surface damaged.

### 2.5. Statistical Analysis

The results were expressed as the mean ± standard error (SE) and subjected to analysis of variance (ANOVA) tests. Significant differences (*p* < 0.05) were determined by a comparison of means conducted with the Tukey’s HSD test. Distinct lowercase letters denote significant differences among treatments for the same sampling period. All statistical evaluations were determined using the SPSS software package, version 22 (IBM Corp.; Armonk, NY, USA).

## 3. Results and Discussion

### 3.1. Respiration Rate, Weight Loss, and Fruit Firmness Evolution

**Figure 1:**
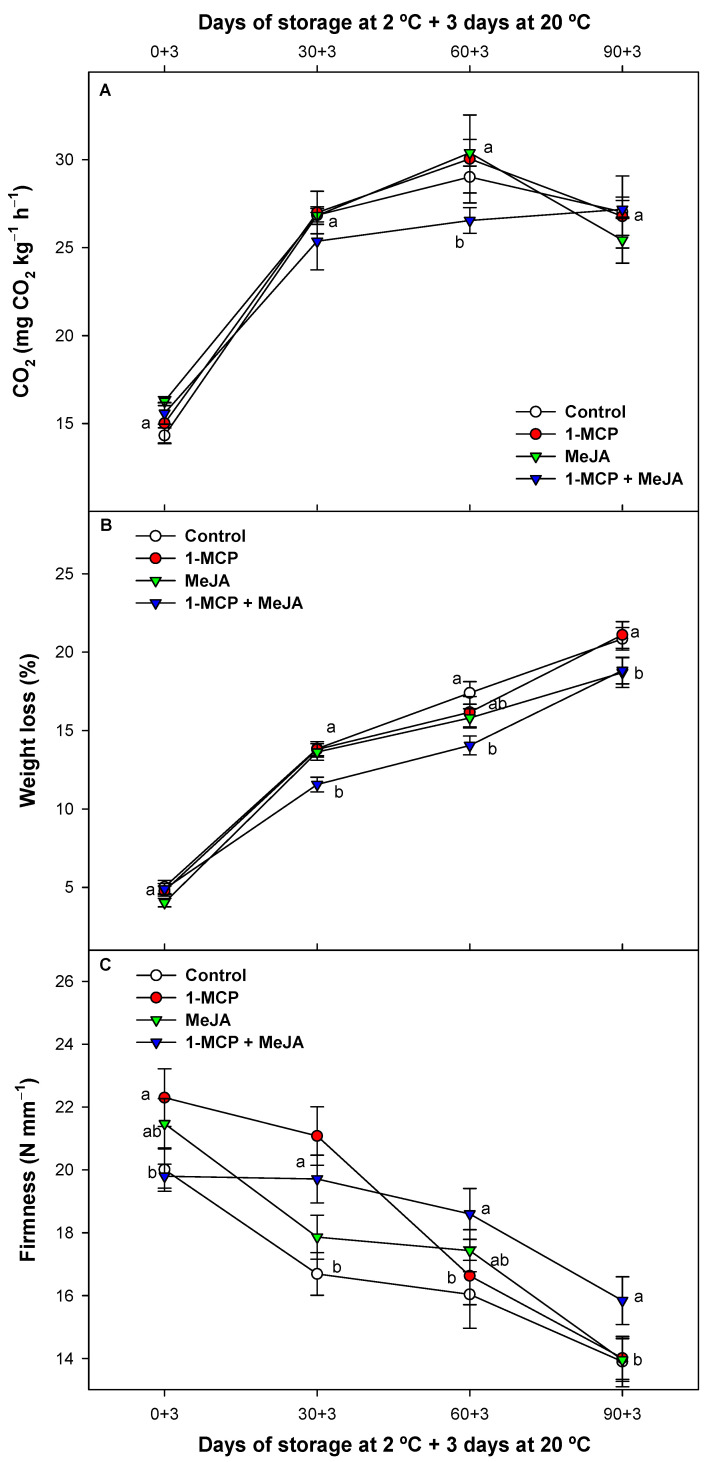
Respiration rate (mg CO_2_ kg^−1^ h^−1^) (**A**), weight loss (%) (**B**), and fruit flesh firmness (N mm^−1^) (**C**) of “Mollar de Elche” pomegranate fruit after treatment with 1-methylcyclopropene (1-MCP), methyl jasmonate (MeJA), and 1-methylcyclopropene + methyl jasmonate (1-MCP + MeJA) postharvest during refrigerated storage at 2 °C plus 3 days at room temperature. Different displayed lowercase letters indicate significant differences (*p* < 0.05) among treatments on the same sampling date. Data are the mean ± SE (*n* = 3).

After cold storage, pomegranates increase their respiration rate at room temperature (Figure 1A). Compared with control fruit, pomegranates treated only with 1-MCP or with MeJA displayed a similar pattern, with no significant differences (*p* > 0.05) between batches. However, respiration was significantly (*p* < 0.05) reduced when 1-MCP and MeJA were applied as a combined treatment during cold storage plus 3 days at 20 °C. At the end of the storage period, the different postharvest treatments did not show any significant differences (*p* > 0.05).

Although 1-MCP and MeJA treatments assayed alone did not result in a decrease in metabolic activity, the favorable impact arising from the combination of these two distinct substances could be attributed to a synergistic effect of their respective properties. In this sense, both substances reduced the metabolism in other non-climacteric fruits such as cherries [30,31], apples and pineapples [32,33], or pomegranates [5,16,17] while preserving cellular membrane integrity and reducing fruit weight loss. In this study, weight loss in the pomegranate batches increased as expected during refrigerated storage (Figure 1B), but pomegranates treated with 1-MCP + MeJA showed a significantly (*p* < 0.05) delayed evolution compared with the other batches studied during the whole experiment. In this sense, in coincidence with respiration, a synergistic effect was observed with the combined application (1-MCP + MeJA) compared with these treatments when applied separately. Pomegranates treated with 1-MCP and control fruit showed the highest dehydration at the last evaluation assayed (90 days of cold storage). The increased fruit weight loss in the experiment may be due to fruit transpiration and respiration processes. In other previous studies, postharvest treatments based on MeJA and 1-MCP applied separately led to reduced pomegranate weight loss [5,10,16,17]. Fruit firmness decreased during cold storage as expected (Figure 1C), and pomegranates treated with 1-MCP maintained a greater fruit firmness compared with control pomegranates during the first storage period. However, during the last part of the cold storage period, greater firmness was obtained by combining treatments (1-MCP + MeJA), thus maintaining this effect during storage. The synergistic effect displayed by combining both technologies may be attributed to their enhanced performance when used together. 1-MCP delays senescence by blocking basal ethylene action in pomegranate fruit [27,34], while MeJA increases the cell energy status [35] and stimulates stress responses and the antioxidative capacity [36]. We previously reported the MeJA effect, when applied at preharvest or as a postharvest treatment, in increasing antioxidative activity and the bioactive compound content of total polyphenols, anthocyanins, or vitamin C in pomegranate fruit [16,17]. This trend was consistent with recent observations reported for minimally processed arils [18], which have been linked to a positive effect in increasing resilience to membrane peroxidation in many fruit species [36,37], resulting in decreased weight loss and the preservation of fruit firmness.

### 3.2. Pomegranate External and Internal Color

**Figure 2:**
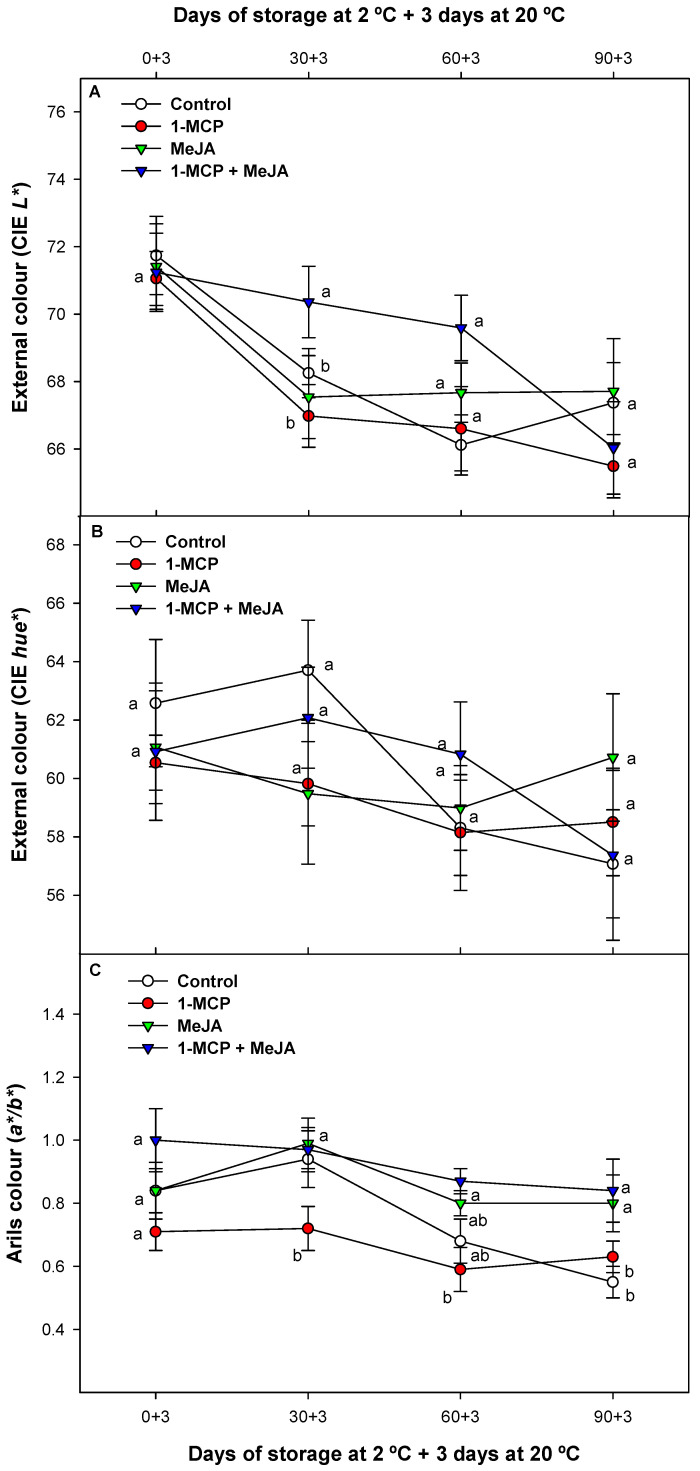
CIE L* (**A**) and CIE hue* (**B**) evolution in the skin and CIE *a*/*b* (**C**) evaluated in arils of “Mollar de Elche” pomegranates treated with 1-methylcyclopropene (1-MCP), methyl jasmonate (MeJA), and 1-methylcyclopropene + methyl jasmonate (1-MCP + MeJA) at postharvest during refrigerated storage at 2 °C plus 3 days at 20 °C. Different lowercase letters indicate significant differences (*p* < 0.05) among treatments for each sampling date. Data are the mean ± SE (*n* = 3).

Pomegranate color is a key indicator of its quality and nutritional content. Aril and husk colors are determined by the amount of anthocyanin pigments, which can vary depending on factors such as variety, growing conditions, and ripeness [38]. CIE L* in pomegranate husk decreased in all samples during storage conditions, with an approximate 5% reduction in all treatments after 90 days of storage. 1-MCP + MeJA was the postharvest treatment which displayed the highest CIE L* values, compared with the remaining fruit batches, which showed significant differences (*p* < 0.05) up to two months of storage. However, these differences were not observed between the different batches after 3 months at 2 °C plus 3 days at 20 °C (Figure 2A). The delayed CIE L* evolution has been linked to a lower rate of fruit weight loss and a delayed postharvest ripening [39] since reduced water evaporation in pomegranate fruit preserves their freshness for longer periods. The CIE hue* angle of the pomegranate external rind also decreased during storage, with no differences (*p* > 0.05) observed over the whole study (Figure 2B).

CIE hue* is an important parameter that provides an objective measure of the fruit color; lower values are related to darker colors [38]. On the contrary, significant differences (*p* < 0.05) were observed in pomegranate arils after measuring the CIE a*/b* ratio, since 1-MCP batches displayed the lowest CIE a*/b* ratio in pomegranate arils, which correlates with a paler aril color [40] (Figure 2C). Aril color evolution was stimulated by MeJA postharvest treatment alone or in combination with 1-MCP, generally with similar values (*p* > 0.05) between them. In this sense, both technologies increased the reddish color of arils significantly (*p* < 0.05) compared with control pomegranates or with 1-MCP treatment applied alone. The deeper red color observed in MeJA-treated fruit alone or in fruit treated with MeJA combined with 1-MCP is related to an increased level of bioactive compounds, such as anthocyanins, as previously reported [16,17,18] and in the present study, as discussed below.

### 3.3. Total Soluble Solids (TSS) and Titratable Acidity (TA)

**Figure 3:**
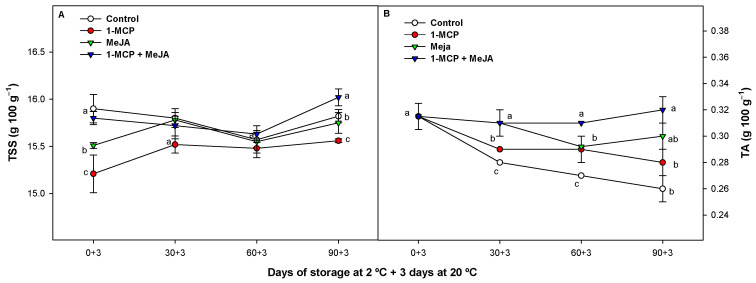
Total soluble solids (TSS) (g 100 g^−1^) (**A**) and titratable acidity (TA) (g 100 g^−1^) (**B**) in aril juice of “Mollar de Elche” pomegranate fruit treated with 1-methylcyclopropene (1-MCP), methyl jasmonate (MeJA), and 1-methylcyclopropene + methyl jasmonate (1-MCP + MeJA) at postharvest during cold storage at 2 °C plus 3 days at 20 °C. Different lowercase letters indicate significant differences (*p* < 0.05) among treatments for each sampling date. Data are the mean ± SE (*n* = 3).

During storage, the amount of TSS showed a slight increase in all samples for up to 90 days. Notably, the 1-MCP + MeJA batch exhibited the highest TSS values, while the 1-MCP batch had the lowest. Specifically, after 90 days, the TSS content in the 1-MCP + MeJA batch was significantly higher (*p* < 0.05) compared with the control fruit. Conversely, the 1-MCP-treated batch displayed significantly lower values (*p* < 0.05) compared with the rest of the treatments evaluated (Figure 3A).

On the other hand, the TA in all the pomegranate batches slightly decreased during the study (Figure 3B). Fruit treated with 1-MCP + MeJA displayed higher TA values compared with control pomegranates after periods of 30, 60, and 90 days (13.84, 15.99, and 19.07% higher, respectively). MeJA and 1-MCP applied separately also delayed TA evolution compared with control fruit, with MeJA batches displaying the greater effectiveness. These modifications occur as part of the ordinary maturation process of pomegranates as previously reported [4,41], characterized by a decrease in TA in the pomegranate juice and an increase in TSS values (mainly sugars). In relation to our results, other authors reported that MeJA-treated arils showed effectiveness in reducing the loss of organic acids such as vitamin C [18] as well as in reducing pomegranate respiration [16,17]. In this sense, 1-MCP can reduce the metabolic activity of pomegranate fruit, delaying senescence [5,27]. These events, coincident with our results, could be related to the enhanced effect observed in maintaining the TSS content and TA when 1-MCP and MeJA were applied as a combined treatment.

### 3.4. Effect of Postharvest Treatments on Total Bioactive Compounds

**Figure 4:**
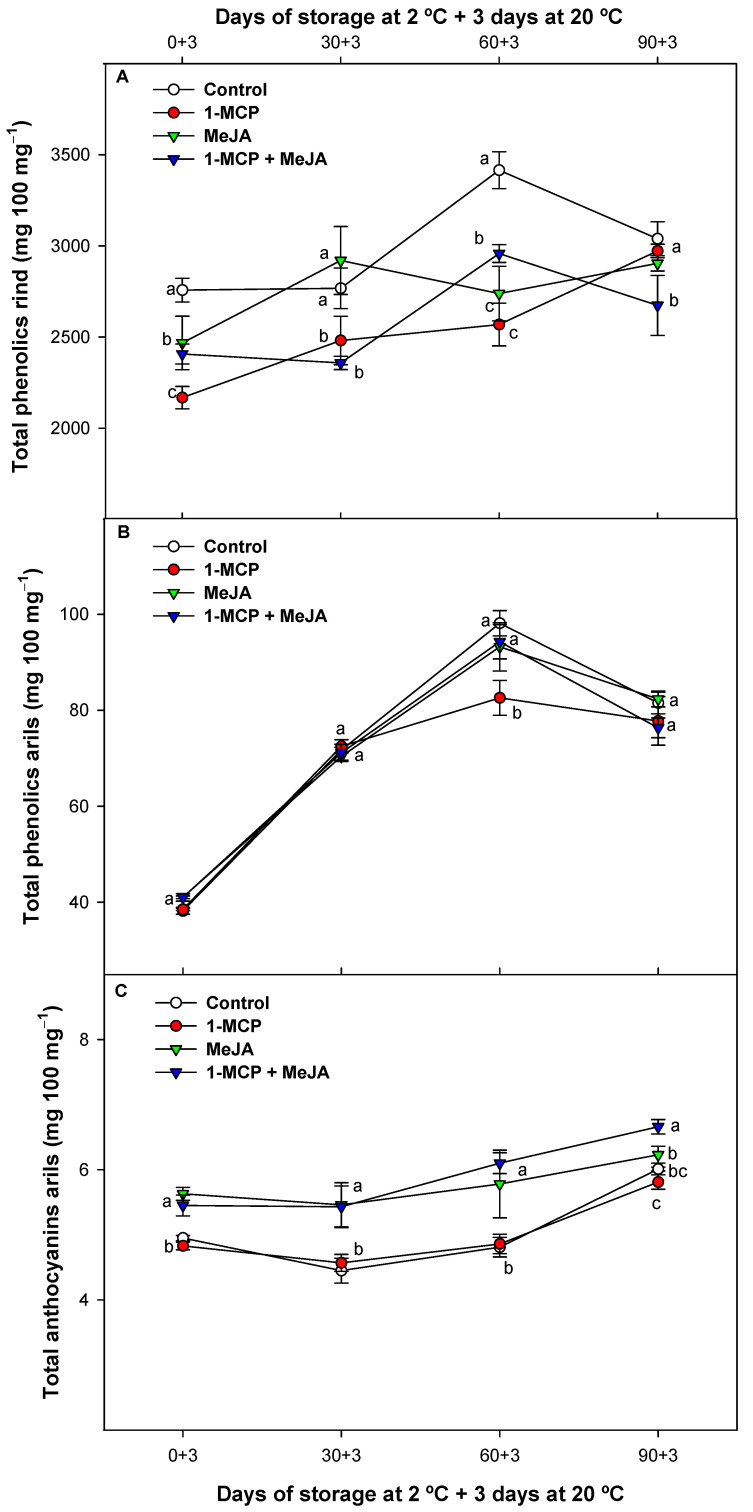
Total phenolic content in rind (mg 100 mg^−1^) (**A**), total phenolic content in arils (mg 100 mg^−1^) (**B**) and total anthocyanin content in arils (mg 100 mg^−1^) (**C**) of “Mollar de Elche” pomegranate fruit treated with 1-methylcyclopropene (1-MCP), methyl jasmonate (MeJA), and 1-methylcyclopropene + methyl jasmonate (1-MCP + MeJA) at postharvest during cold storage at 2 °C plus 3 days at 20 °C. Different lowercase letters indicate significant differences (*p* < 0.05) among treatments for each sampling date. Data are the mean ± SE (*n* = 3).

Consumers value pomegranates for the presence of bioactive compounds with antioxidative attributes [16]. These phytochemical compounds are mainly phenolic substances, such as anthocyanins and other flavonoids, as well as hydrolysable tannins such as ellagitannins, gallic acid, and ellagic acid. These constituents have been documented to contribute to the health-promoting qualities associated with the consumption of pomegranate, offering protection against certain degenerative diseases [2,3]. The concentration of total polyphenols in the pomegranate rind increased during storage (Figure 4A). The highest values were observed in control fruit compared with the other treatments, whether combined or separately applied. On the other hand, the polyphenol content in arils showed an initial upward trend in all treatments during the first 60 days of cold storage, followed by a subsequent decline, probably because of the degradation stimulated by polyphenol oxidase [18]. The evolution of these bioactive compounds was delayed only for the 1-MCP-treated fruit batch (≈83–100 mg^−1^) after 60 days of refrigerated storage compared with the rest of batches (≈96–98 mg 100 mg^−1^) (Figure 4B). The total anthocyanin content slightly increased in all pomegranates during storage (Figure 4C). However, treatments containing MeJA elicited an approximately 20% increase in these red-colored compounds. The effect was maintained through the whole storage period with significantly (*p* < 0.05) higher values for the combined treatment (1-MCP + MeJA) at the end of the study. MeJA application by itself increased the red coloration in pomegranate arils during ripening on-tree and after harvest during storage [16,17,18]. Additionally, in previous studies, 1-MCP has shown a positive effect reducing the polyphenol oxidase (PPO) activity in pomegranate fruit [5]. This could explain the higher anthocyanin content in the combined treatment since MeJA stimulates anthocyanin accumulation, while 1-MCP could be preventing the PPO oxidative action.

### 3.5. Membrane Permeability (MDA Content and EL) and Chilling Injury (CI)

**Figure 5:**
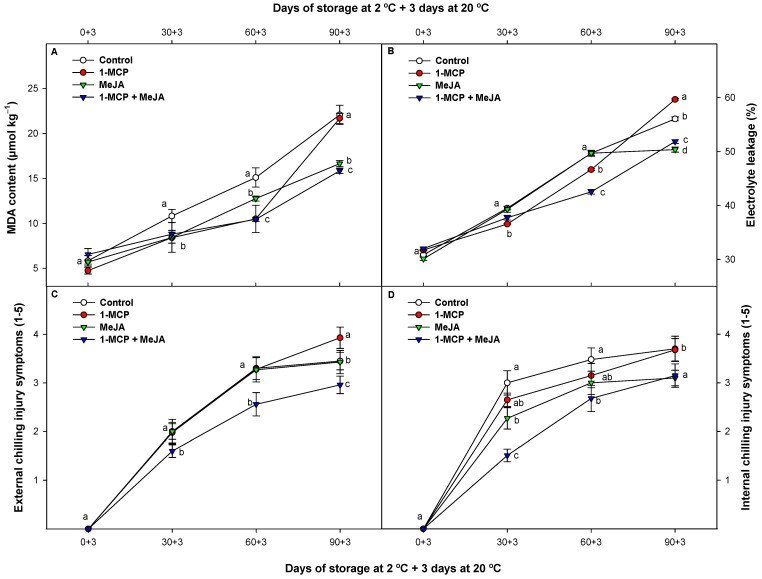
Evolution of malondialdehyde (MDA) content (μmol kg^−1^) (**A**), electrolyte leakage (EL) (%) (**B**), and chilling injury symptoms in the external (**C**) and internal (**D**) peel of “Mollar de Elche” pomegranate fruit treated with 1-methylcyclopropene (1-MCP), methyl jasmonate (MeJA), and 1-methylcyclopropene + methyl jasmonate (1-MCP + MeJA) at postharvest during cold storage at 2 °C plus 3 days at 20 °C. Data are the mean ± SE (*n* = 3). Different lowercase letters indicate significant differences (*p* < 0.05) among treatments for each sampling date.

Aldehydes are some of the most important products obtained after lipid peroxidation and can be indicators of catabolism. The main aldehyde compound is MDA [42]. MDA increased in all samples during cold storage. Pomegranate fruit after treatments based on 1-MCP, MeJA, or 1-MCP + MeJA showed a reduction of 22.45, 22.17, and 18.75%, respectively, after 60 days at 2 °C plus 3 more days at 20 °C. The MDA content in control fruit was, in general, significantly higher (*p* < 0.05) compared with the other pomegranate batches during the experiment. However, the MDA evolution was delayed when MeJA was applied alone compared with when 1-MCP was assessed. Moreover, pomegranate fruit treated with 1 MCP + MeJA displayed the strongest effect in reducing MDA content compared with the rest of the pomegranate batches at the end of the study (Figure 5A).

**Figure 6:**
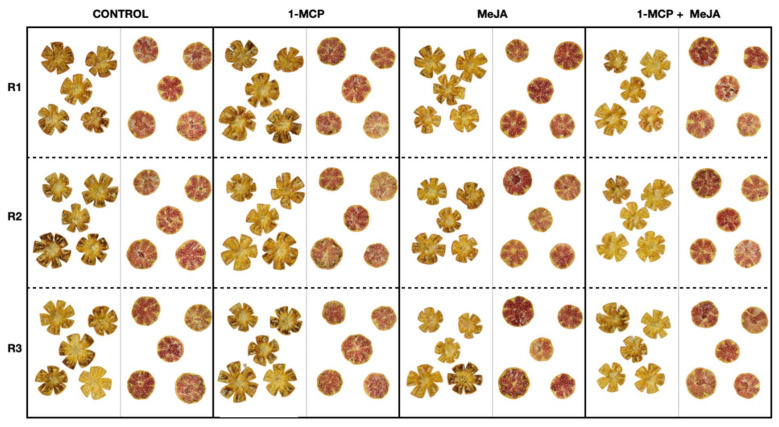
Internal visual aspect (mesocarp and arils) of pomegranates treated with 1-methylcyclopropene (1-MCP), methyl jasmonate (MeJA), and 1-methylcyclopropene + methyl jasmonate (1-MCP + MeJA) at postharvest after 30 days of cold storage at 2 °C plus 3 days at 20 °C.

High concentrations of MDA and other free radicals are poisonous in plant tissues, leading to cell and lipid membrane peroxidation. In this sense, EL indicates changes in membrane integrity during refrigerated storage [43]. In the husk tissues, EL increased during this study, although the EL was delayed significantly (*p* < 0.05) in 1-MCP batches and in pomegranates treated with the combined application (1-MCP + MeJA) compared with control pomegranates after 30 days of storage (Figure 5B). After this period, untreated and 1-MCP-treated batches exhibited higher EL levels (8.09 and 15.05%, respectively) than the 1-MCP + MeJA batch. Earlier studies have indicated that 1-MCP and MeJA may stimulate the activity of antioxidative enzymes in pomegranate [5,44] and other non-climacteric fruit [45], resulting in a reduction in MDA content and a decrease in EL, which is consistent with the results obtained in this study. Consequently, the positive effect in reducing the MDA content and EL when 1-MCP or MeJA were used individually might explain why the delay observed in the evolution of the MDA content and EL were more effective when these substances were applied together (1-MCP + MeJA). On the other hand, as expected, pomegranate fruit were susceptible to CI at 2 °C, displaying symptoms of this disorder on the external and internal rinds after 30 days of storage at 2 °C plus 3 more days at 20 °C (Figure 5C and Figure 6). MeJA or 1-MCP treatments, when administered individually, did not show efficacy in delaying external CI compared to control fruit, except toward the end of cold storage, where both compounds demonstrated a reduction in this parameter (Figure 5C). Nonetheless, the application of these treatments as a combined treatment throughout the entire storage period at suboptimal temperatures resulted in a significant (*p* < 0.05) delay in external CI. On the other hand, the photographs prominently illustrate the discernible impact of different treatments on internal CI (Figure 6). Also, in these photographs, the effect of 1-MCP in delaying aril color development can be observed, as well as the contrary effect displayed in MeJA-treated pomegranate alone or MeJA combined with 1-MCP.

CI in the mesocarp (internal CI) showed similar results to that previously observed for the epicarp (Figure 5D). However, internal CI was delayed not only with the combination of treatments (1-MCP + MeJA) but also in 1-MCP and especially in MeJA-treated fruit, which showed a significant cold tolerance compared with control batches. CI developed after removing the fruit from suboptimal temperatures to room temperature. Even when applied individually or in combination, neither treatment was able to completely prevent CI at suboptimal temperatures such as 2 °C. It is reasonable to assume, however, that due to the substantial delay in the onset of this damage, these technologies might be highly effective in postponing the occurrence of such damage at higher cooling temperatures. The main indicators of CI include rind desiccation, browning, pitting, surface depressions, and an increased vulnerability to decay [46]. The progression of CI is related to an elevation in membrane permeability due to modifications in lipids and proteins [47]. In this sense, 1-MCP and MeJA have shown enhanced resilience to CI in other non-climacteric species when applied alone as both these different substances lead to a common outcome in pomegranate fruit as has been displayed in this study: both of them applied alone improved membrane integrity, reducing EL and the MDA content, which is related to the extended functionality and maintenance of cellular tissues [25,36]. Additionally, MeJA treatments provide an increased energy status coincident with stress events (such as senescence or storage at suboptimal temperatures), thus reducing metabolic demands and respiration [35]. On the other hand, 1-MCP is known to reduce the basal ethylene impact on pomegranates [25], thus stimulating respiration and different metabolic pathways [34]. These events involve a multifactorial metabolic system that could be linked to the synergistic effect of 1-MCP + MeJA in reducing the MDA content, EL, and CI under suboptimal temperatures, reducing the respiration process.

## 4. Conclusions

The data presented in this study suggest that methyl jasmonate vapor on pomegranate alleviates chilling injury and reduces membrane disintegration while also augmenting the anthocyanin content. Weight loss and fruit softening were delayed with the different strategies applied in this study, maintaining the total acidity. The accumulation of total phenolic compounds was delayed, but the total anthocyanin content was stimulated with strategies containing methyl jasmonate. Membrane permeability was reduced, delaying chilling injury. The methyl jasmonate effect in reducing chilling injury and delaying quality losses was higher than that observed with 1-methylcyclopropene applications. Fruit firmness and membrane stability were well controlled by 1-methylcyclopropene, leading to a delay of senescence. However, the best results in terms of fruit quality were observed when both strategies were combined. Different pomegranate cultivars may respond differently to these treatments, particularly under suboptimal storage temperatures. The different strategies assayed in this study did not completely prevent the chilling injury in pomegranate fruit. On the other hand, the results suggest that pomegranate storability at higher cooling temperatures could be enhanced by combining these different postharvest treatments, but further studies will be necessary to elucidate the limitations of these technologies based on the storage temperature. However, the observed impact of these strategies, which can be simultaneously applied, could be a new promising approach to control chilling injury and fruit senescence during cold storage in pomegranate fruit and other fruit species.

## Data Availability

Data is contained within the article.
